# Editorial: Biophysics approaches to investigate multi-organ alcohol-induced damage

**DOI:** 10.3389/fmolb.2023.1346518

**Published:** 2023-12-18

**Authors:** Janos Paloczi, Youngchan Kim

**Affiliations:** ^1^ Department of Physiology, Louisiana State University Health Sciences Center, New Orleans, LA, United States; ^2^ Department of Microbial Sciences, School of Biosciences, University of Surrey, Guilford, United Kingdom; ^3^ Leverhulme Quantum Biology Doctoral Training Centre, University of Surrey, Guildford, United Kingdom; ^4^ Advanced Technology Institute, University of Surrey, Guildford, United Kingdom

**Keywords:** alcohol research, alcohol misuse, alcohol induced disorders, alcohol binge drinking, biophysical approach

Alcohol remains the most commonly used intoxicant globally, particularly among young individuals. Its easy accessibility has led to extensive personal and social use, particularly during the recent years of the pandemic. Alcohol consumption can result in a spectrum of diseases affecting various organs in alcohol-dependent individuals, resulting in a complex disease profile and severe consequences. According to the latest report from the World Health Organization on the global status of alcohol and health, alcohol has been responsible for nearly 3 million deaths annually in recent years, or around 6 deaths per minute worldwide. This underscores the urgent need for new measures to alleviate the burden of alcohol-related diseases.

The application of a multi-omics approach in alcohol research has recently emerged as a powerful and comprehensive strategy to disentangle the complex interplay of molecular events underlying alcohol-induced physiological responses. The latest advances in the field of alcohol research aim to integrate genomics, transcriptomics, proteomics, metabolomics, and epigenomics to enhance our understanding of the development and progression of alcohol-induced diseases (Kapoor et al., 2021; Zillich et al., 2023; Mavromatis et al., 2022; Lohoff et al., 2022). This systems biology approach allows us to identify key biomarkers, pathways, and molecular signatures associated with alcohol-related conditions, such as liver disease, cardiovascular dysfunction, and neurological disorders. The comprehensive analysis of these omics layers enables the identification of potential therapeutic targets and personalized interventions, facilitating a more nuanced and precise approach to alcohol-related health challenges. Moreover, the integration of multi-omics data enhances our ability to comprehend the intricate relationships between genetic predisposition, environmental factors, and alcohol-induced pathophysiology contributing to a more profound insight into the complexities of alcohol-related disorders.

Our Research Topic, “*Biophysics Approaches to Investigate Multi-organ Alcohol-induced Damages*,” featured newly developed tools in alcohol research. It combined multi-omics-based approaches, structural biology, biochemistry, biophysics, and translational aspects to study the effects of excessive alcohol misuse. Our ultimate goal was to provide a comprehensive understanding of the molecular mechanisms underlying alcohol addiction and alcohol-induced organ damage from the biophysicist’s perspective. Through the use of cutting-edge approaches, we aimed to identify potential therapeutic targets for treating alcohol use disorder.

This Research Topic includes original research papers as well as reviews that offer a unique insight into the biophysical characteristics of alcohol misuse, with potential applications for alcohol research.

• In a comprehensive review, Lee and Kim summarized our current understanding of how plant-derived activators of nuclear receptors, specifically isoflavones, can serve as a novel therapeutic strategy to mitigate alcoholic liver disease. They highlighted their potential to improve hepatocyte survival and attenuate liver damage through the regulation of macrophages and other immune cell types. The authors also emphasized the significance of computational approaches as valuable tools for studying the molecular interactions of isoflavone analogues with their targets.
• Another review by Shortall et al. focused on the combined examination of the structural, functional, and biophysical properties of bacterial and eukaryotic alcohol dehydrogenases (ALDHs), including their roles in human disease.
• Shalchi-Amirkhiz et al. demonstrated that a single session of binge alcohol drinking does not significantly alter the biophysical features of leukocytes. However, *in vitro* exposure of leukocytes to ethanol during stimulation inhibits the cytoskeleton reorganization of monocytes and neutrophils, impeding cell deformability and thereby affecting immune cell functions.
• Chen et al. reported that plasma levels of homocysteine are not significantly associated with three non-alcoholic fatty liver-related diseases (non-alcoholic fatty liver disease or its progression, nonalcoholic steatohepatitis, and cirrhosis) as revealed by the two-sample Mendelian Randomization method.
• Singh et al. and co-workers investigated whether polymorphisms in the major alcohol-metabolizing enzyme genes (alcohol dehydrogenase (ADH), aldehyde dehydrogenase (ALDH), and cytochrome P450 enzymes) could be linked to alcoholic pancreatitis. They concluded that individuals carrying the ADH3*1/*1 allele and consuming alcohol are at a higher risk for alcoholic pancreatitis than those with other genotypes of the ADH enzyme.

In conclusion, exploring the biophysical aspects of alcohol research provides a crucial dimension to our understanding of its impact on the human body. The authors contributing to this Research Topic employed cutting-edge tools and methodologies gaining a unique perspective on how alcohol misuse affects cellular and physiological functions. This in-depth exploration not only enhances our ability to mitigate the burden of alcohol-related diseases but also lays the groundwork for more targeted and effective interventions in the realm of addiction and public health.
